# Chromophore binding to two cysteines increases quantum yield of near-infrared fluorescent proteins

**DOI:** 10.1038/s41598-018-38433-2

**Published:** 2019-02-12

**Authors:** David Buhrke, Neslihan N. Tavraz, Daria M. Shcherbakova, Luisa Sauthof, Marcus Moldenhauer, Francisco Vélazquez Escobar, Vladislav V. Verkhusha, Peter Hildebrandt, Thomas Friedrich

**Affiliations:** 1 Institut für Chemie, Sekr. PC14, Technische Universität Berlin, Straße des 17. Juni 135, 10623 Berlin, Germany; 2 Department of Anatomy and Structural Biology, Albert Einstein College of Medicine, 1300 Morris Park Avenue, Bronx, NY 10461 USA; 3 Charité – Universitätsmedizin Berlin, Institute of Medical Physics and Biophysics (CC2), Group Protein X-ray Crystallography and Signal Transduction, Charitéplatz 1, 10117 Berlin, Germany

## Abstract

Phytochromes are red/far-red light sensing photoreceptors employing linear tetrapyrroles as chromophores, which are covalently bound to a cysteine (Cys) residue in the chromophore-binding domain (CBD, composed of a PAS and a GAF domain). Recently, near-infrared (NIR) fluorescent proteins (FPs) engineered from bacterial phytochromes binding biliverdin IXα (BV), such as the iRFP series, have become invaluable probes for multicolor fluorescence microscopy and *in vivo* imaging. However, all current NIR FPs suffer from relatively low brightness. Here, by combining biochemical, spectroscopic and resonance Raman (RR) assays, we purified and characterized an iRFP variant that contains a BV chromophore simultaneously bound to two cysteines. This protein with the unusual double-Cys attached BV showed the highest fluorescence quantum yield (FQY) of 16.6% reported for NIR FPs, whereas the initial iRFP appeared to be a mixture of species with a mean FQY of 11.1%. The purified protein was also characterized with 1.3-fold higher extinction coefficient that together with FQY resulted in almost two-fold brighter fluorescence than the original iRFP as isolated. This work shows that the high FQY of iRFPs with two cysteines is a direct consequence of the double attachment. The PAS-Cys, GAF-Cys and double-Cys attachment each entails distinct configurational constraints of the BV adduct, which can be identified by distinct RR spectroscopic features, i.e. the marker band including the C=C stretching coordinate of the ring A-B methine bridge, which was previously identified as being characteristic for rigid chromophore embedment and high FQY. Our findings can be used to rationally engineer iRFP variants with enhanced FQYs.

## Introduction

Phytochromes constitute a class of bimodal photoreceptor proteins, which incorporate linear tetrapyrrole cofactors for red light sensing in plants, fungi, cyanobacteria and non-photosynthetic bacteria^1^. Bacterial phytochromes that incorporate biliverdin IXα (BV) have been extensively used for engineering of fluorescent proteins (FPs)^2–6^. The two advantages of these FPs, their near-infrared (NIR) spectra and the presence of the BV chromophore in mammalian tissue, made them useful probes for non-invasive whole-body imaging and multicolor fluorescence microscopy^7,8^. However, all current NIR FPs suffer from relatively low molecular brightness with the fluorescence quantum yield (FQY) varying from 6–14%. Recent studies found that chromophore attachment in the brightest of engineered NIR FPs differs from the canonical BV binding found in wild-type bacterial phytochromes^9–11^.

**Figure 1:**
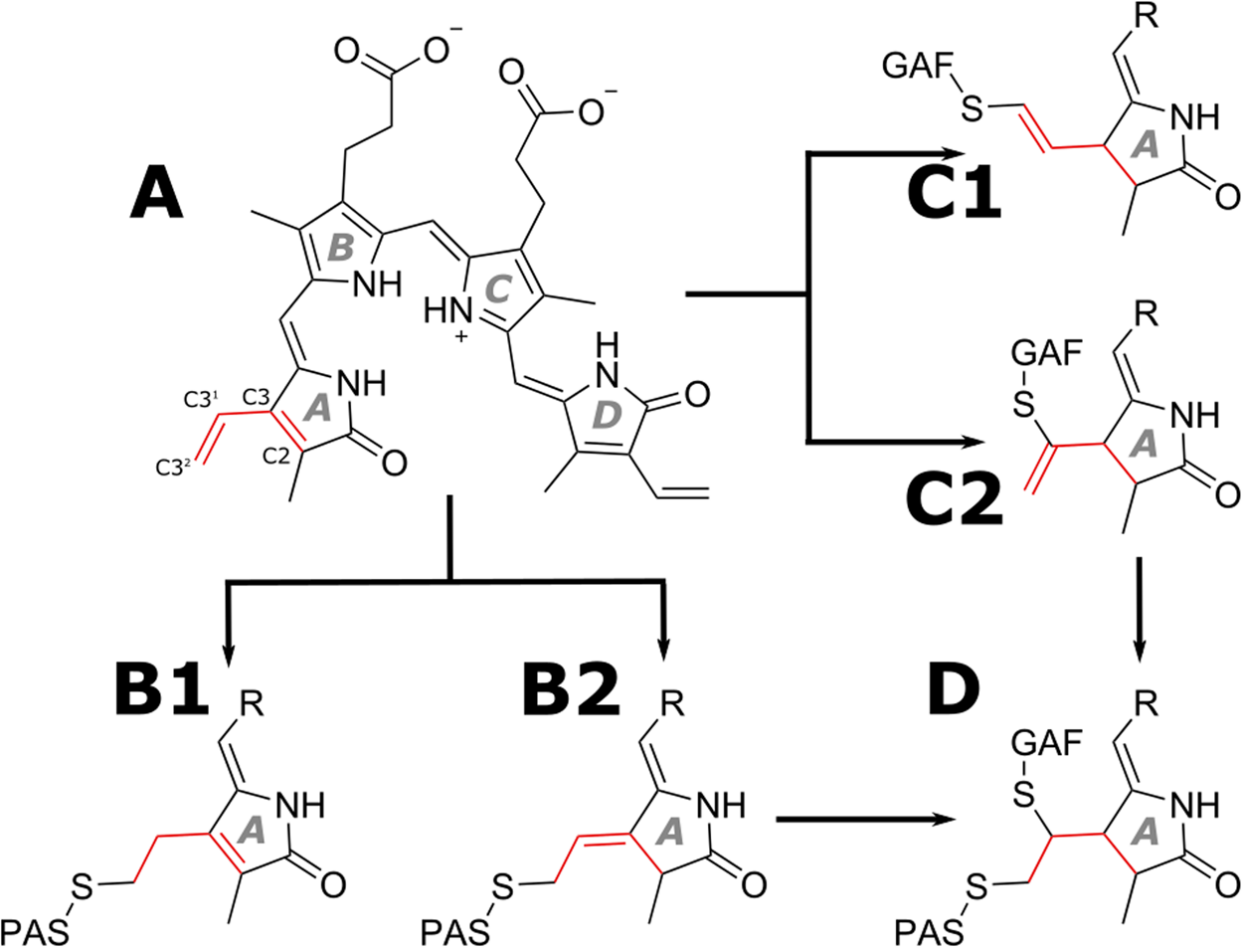
Possible modes of chromophore attachment. Free BV (**A**) can be covalently attached to the conserved PAS-Cys under retention of the endocyclic DB (**B1**^24^) or exocyclic DB rearrangement (**B2**^19^), as found in the crystal structures of various BphPs. Attachment to the GAF-Cys results in blue-shifted absorption properties and the remaining DB is not conjugated to the aromatic system, accordingly. Hence, attachment to the GAF domain either results in configuration (**C2**)^9^ like in eukaryotic phytochromes^17^ (albeit with a non-conjugated DB) or, possibly also in the configuration (**C1**), as found in miRFP670^11^. Upon further reaction, only the structures (**B2** and **C2**) can lead to the reported structure of the double-attached species (**D**), while (**B1**,**C1**) represent dead ends in the reaction sequence.

In all phytochromes, an open-chain linear tetrapyrrole chromophore is covalently conjugated to a cysteine (Cys) residue in the chromophore-binding domain (CBD) via a thioether bridge in an autocatalytic process termed autolyase activity^12^. Although this fundamental reaction is conserved in all known phytochromes, the chromophore and the attachment site are different in bacterial and plant variants. In the case of bacterial phytochromes (BphPs), biliverdin IXα (BV) is attached to a conserved Cys residue upstream of the PAS domain (denoted PAS-Cys in the following)^13^, whereas in cyanobacterial and plant phytochromes the corresponding phycocyanobilin (PCB) or phytochromobilin (PΦB) cofactors are linked to a Cys in the GAF domain^14^. Ring A of PCB and PΦB contains one exocyclic double bond (DB) at the ethylidene substituent, but no endocyclic DB, and an unambiguous mode of attachment to the GAF-Cys is found in the cyanobacterial phytochromes Cph1^15^, Cph2^16^ and in plant phytochrome B^17^. In contrast, ring A of free BV contains an endocyclic DB and a second DB in the vinyl group (Fig. 1, structure A). Consequently, the configuration of the remaining DB in BV after attachment to bacterial phytochromes can vary. In the bacterial phytochrome Agp1^18^, preservation of the endocyclic ring A DB was observed (Fig. 1, structure B1), but investigation of the highly homologous DrBphP revealed an exocyclic DB rearrangement (Fig. 1, structure B2)^19^. Another way of chromophore binding was found in engineered proteins derived from bacterial phytochromes. The first evidence that chromophore binding is possible to the Cys in the GAF domain of BphPs was detected for a Cys exchange mutant of DrBphP that covalently binds PCB via attachment to a GAF-Cys^19^. Later, covalent binding of BV chromophore to the Cys residue in the GAF domain was discovered in blue-shifted near-infrared fluorescent proteins^9^. The resulting chromophore structures are shown in Fig. 1 (structures C1 and C2). In these structures, the DB between the C3^1^ and C3^2^ atoms is out of conjugation with the rest of the π-electron system, which results in a spectral blue shift in NIR fluorescent proteins containing a Cys in the GAF domain.

**Figure 2:**
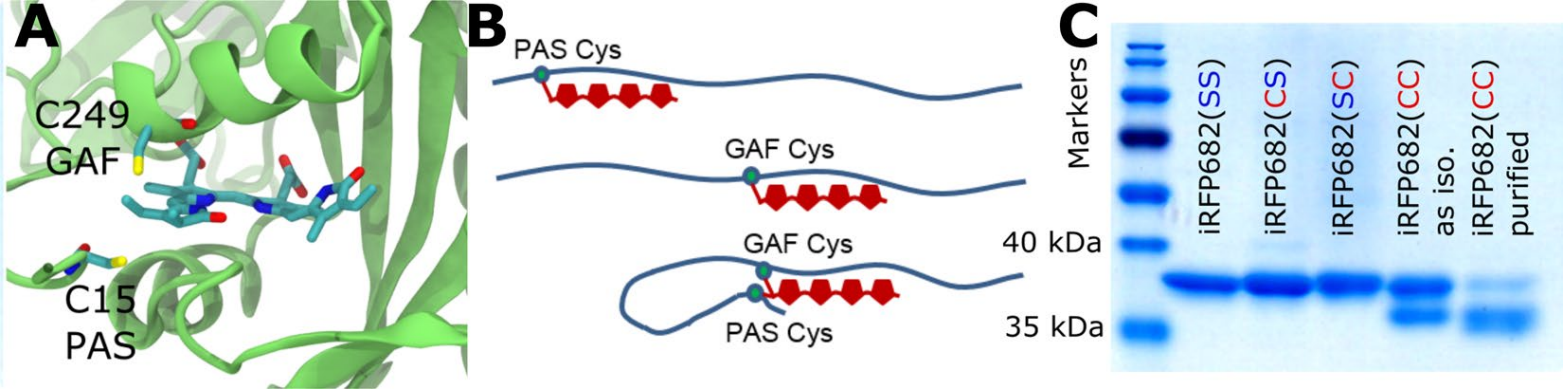
Double-Cys chromophore attachment in iRFP682. (**A**) The positions of the PAS- and GAF-Cys (C15, C249) residues (exchanged for Ser in mutants) relative to the chromophore in the investigated protein constructs. The presented homology model was derived from the iRFP713 homology model^27^. (**B**) Scheme showing denatured iRFP variant proteins with covalently attached BV. Double attachment results in a covalently linked loop that withstands denaturation and leads to migration of the protein with apparently lower molecular weight. (**C**) Purification of iRFP682(CC). All protein variants containing only one Cys residue ran as a single band in SDS-PAGE at an apparent molecular mass of 37 kDa. iRFP682(CC) displays a second band characteristic for iRFPs with double-Cys motif^10^ at an apparently lower mass, which was assigned to a double attached BV species^11^. This protein species was purified by treatment with GdnHCl and size-exclusion chromatography (Supplementary Fig. S2).

Recently, the crystal structure of miRFP670^20^, an engineered version of the CBD fragment of *Rhodopseudomonas palustris* phytochrome P1 (RpBphP1), was determined^11^. This protein contains two Cys residues in spatial proximity to the vinyl group of the BV ring A (Fig. 2A), one located in the PAS and the other in the GAF domain at homologous positions to the conserved Cys for BphPs, and eukaryotic phytochromes, respectively. The crystallographic data for miRFP670 revealed a novel BV binding mode to a BphP. In addition to a single covalent attachment to the GAF-Cys (Fig. 1, structure C1), a novel double attachment to both Cys residues was observed (Fig. 1, structure D). Attachment to a secondary Cys residue also occurs naturally in diverse cyanobacteriochromes, but in these cases, the secondary Cys is also located at different positions in the GAF domain and adds to the A-B or B-C methine bridges^21,22^.

These findings motivated the present study to determine the binding pattern of BV in iRFP682, another iRFP containing two Cys residues, which was derived from the CBD of RpBphP2^4^. The crystal structure of the prototypical phytochrome precursor RpBphP2 is known^23^, and a structural model of the Cys positions in relation to the embedded BV chromophore is shown in Fig. 2A.

## Results and Discussion

### Mutagenesis and protein biochemistry

Starting from the original construct (denoted iRFP682(CC) in the following) with the Cys residues C15 in the PAS and C249 in the GAF domain (Fig. 2A), we exchanged, first, either of the two cysteines, and second, both cysteines simultaneously for serine, which is non-reactive in chromophore attachment. In this way, the serine exchange variants in the PAS domain (iRFP682(SC)), the GAF domain (iRFP682(CS)) and the all-serine variant (iRFP682(SS)) were obtained. Except for iRFP682(SS), all protein variants bound BV covalently, as confirmed by Zn^2+^ fluorescence (Supplementary Fig. S1) performed in-gel after SDS-PAGE, and a characteristic double-band feature was observed only for iRFP682(CC). Previous observation of this motif for iRFP682^10^ was assigned to a compact cross-linked protein species as a result of double Cys attachment. The resulting topological loop in the protein prevents complete denaturation during SDS treatment, and the protein migrates at a lower apparent mass during electrophoresis. These findings were confirmed by biochemical methods and are in line with corresponding crystallographic data for miRFP670, indicating a mixture of a GAF-only and a double attached species^11^.

### Purification of the double attached species

The two distinct protein species of iRFP682(CC) were not separable by various conventional methods (ion-exchange chromatography, IEC; hydrophobic interaction chromatography, HIC; and size-exclusion chromatography, SEC) due to their identical protein surfaces and, therefore, identical biophysical properties in chromatography experiments. However, iRFP682(CC) showed a spectroscopically heterogeneous denaturation behavior when subjected to high guanidinium hydrochloride (GdnHCl) concentrations. A previous study showed that spectroscopically distinguishable subspecies of iRFP682(CC) denature at different concentrations of GdnHCl. Before the possibility of double-Cys attachment was considered, this behaviour was originally assigned to an allosteric effect caused by dimer-interaction^10^. However, we reasoned that a double-Cys attached and, therefore, cross-linked protein might be more stable under denaturing conditions than the corresponding singly attached variant and could eventually cause this heterogeniety. To confirm that the two species observed in the gel are in fact the reason for the heterogeneous denaturation behaviour, a partially denatured sample (incubation in 2.8 M GdnHCl for 24 h, for detailed protocol on GdnHCl treatment, see Supplementary Fig. S2) was subjected to SEC. After this treatment, a purified sample of a highly enriched double attached species with only minor residual contribution of a singly attached fraction (the densitometric ratio of the gel bands was 4:1) was identified by SDS-PAGE analysis (Fig. 2C, denoted as iRFP682(CC) purified).

### UV/Vis spectroscopy

**Figure 3:**
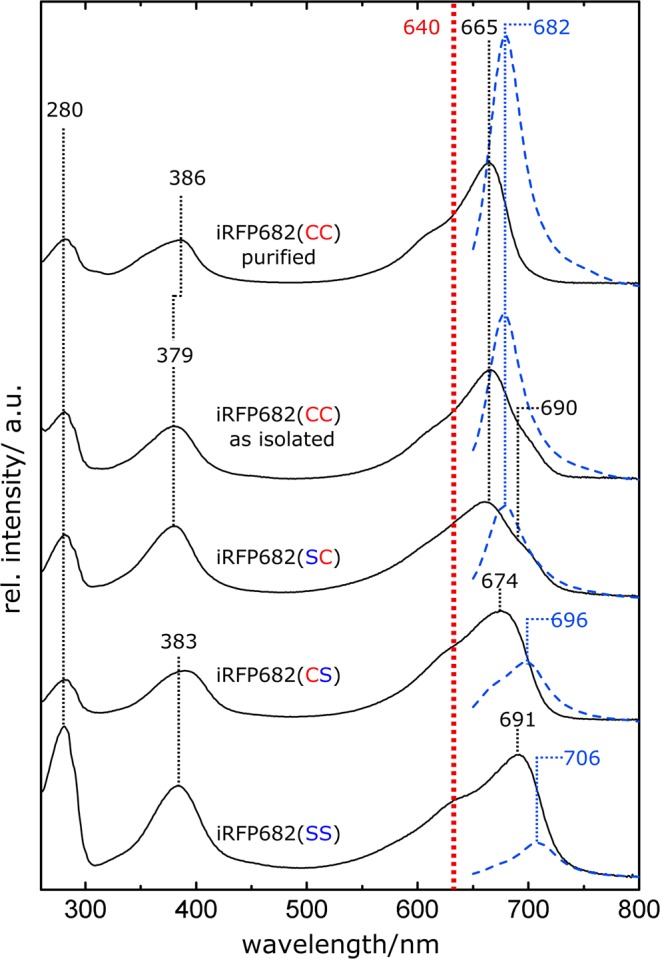
Absorption (black lines) and fluorescence (blue dotted lines) spectra of iRFP682 variants and the purified double attached species. The absorption spectra were normalized at 640 nm (red dotted line), which corresponds the fluorescence excitation chosen for all protein samples. Relative fluorescence intensities were normalized according to the number of absorbed photons at 640 nm.

iRFP682(CS) displays an absorption pattern in the Q-band (max. 674 nm) and the Soret band region (max. at 383 nm) with the characteristic structure and spectral position of BV-binding BphPs in the Pr state (Fig. 3). Compared to iRFP682(CS), the Q-band maximum in the cysteine-deficient variant iRFP682(SS) is red-shifted (691 nm) towards the absorbance maximum of the free BV, which would also be in line with the increased conjugation length (by one DB) of the free chromophore, and the sample contained considerable amounts of apoprotein, indicated by the high relative intensity in the region of protein absorption (280 nm) due to less effective chromophore assembly without covalent attachment. In contrast, iRFP682(SC) shows a blue-shifted absorption maximum of the Q-band (665 nm). Furthermore, the shape of the Q-band is altered in iRFP682(SC) and iRFP682(CC) as isolated, and a shoulder at 690 nm emerges. After GndHCl purification, the shoulder is no longer detected, and iRFP682(CC) displays a homogeneous absorption pattern like PΦB bound to a GAF domain Cys in plant phytochromes (see e.g.^17^). However, in case of iRFP682(CC) the BV chromophore is linked to two Cys residues that should result in a more rigid chromophore embedment.

### Fluorescence spectroscopy

In line with the absorption properties, the fluorescence emission maxima of iRFP682(SS) and (CS) variants are red-shifted (max. 706 and 696 nm), and the intensities are low compared to the other variants. In contrast, iRFP682(SC) and iRFP682(CC) as isolated and purified show the characteristic high-intensity fluorescence peak (max. 682 nm), albeit with different amplitudes. Notably, after the purification procedure, the relative fluorescence intensity of iRFP682(CC) is strongly increased. By integration of the fluorescence signals and extrapolation from the known FQYs of the other variants (CS: 3.2%, SS: 2.2%, CS: 5.0%, CC (as isolated): 11.1%^10^), we estimated an FQY of 16.6% for the purified iRFP682(CC) with double-Cys attachment of the chromophore (Table 1, and Fig. S3). This is the highest FQY reported for the NIR FPs engineered from BphPs so far^8^. This variant also has 1.3-fold increased extinction coefficient. Together, this resulted in almost two-fold increase in molecular brightness.

**Table 1:** Properties of the studied iRFP682 protein species.

iRFP variant	Absorbance max (nm)	Emission max (nm)	Extinction coeffient at absorbance max^a^ (M^−1^·cm^−1^)	Fluorescence quantum yield (%)	Molecular brightness *vs* iRFP682(CC) as isolated^d^ (%)
iRFP682(SS)	691	706	54400	(2.2)^b^	13
iRFP682(CS)	674	696	87400	(3.2)^b^	30
iRFP682(SC)	665	682	57400	(5.0)^b^	31
iRFP682(CC) as isolated	665	682	84400	(11.1)^b^	100
iRFP682(CC) purified	665	682	112200	16.6^c^	199

^a^Molecular extinction coefficients were calculated from the ratio of the Soret and Q-band intensity according previous studies^2^. ^b^Parameters in parentheses refer to the original publication^10^. ^c^The fluorescence quantum yield (FQY) of purified iRFP682(CC) was calculated according to Supplementary Fig. S3. ^d^Molecular brightness is the product of extinction coefficient and FQY.

### Resonance Raman spectroscopy

**Figure 4:**
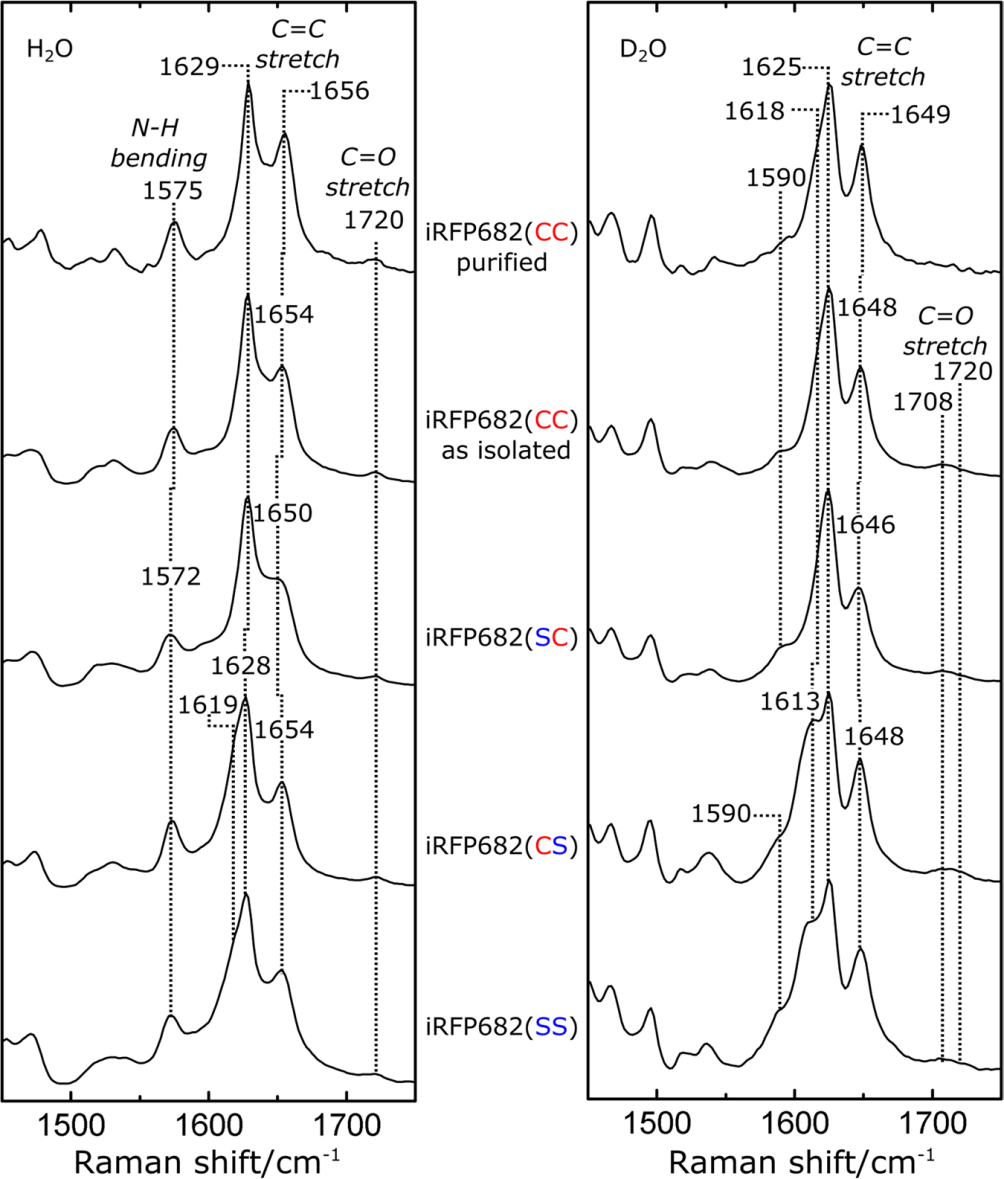
Resonance Raman (RR) spectra of the investigated iRFP constructs in the marker region from 1450 to 1750 cm^−1^. Left panel: spectra recorded in H_2_O buffer, right panel: D_2_O buffer. The characteristic modes corresponding to the C=C and C=O stretching modes as well as the (ring B,C) N-H in-plane bending mode are labeled. Band assignment was performed according to previous studies^25,26^. For full spectra, see Supplementary Fig. S4.

We restrict the discussion to the spectral region comprising the resonance Raman (RR) bands, which are characteristic of specific structural properties of the chromophore (Fig. 4, for full spectral range, see Supplementary Fig. S4). The protonation state of BV is indicated by the band at 1572/1575 cm^−1^. This band originates from the in-phase in-plane bending of the ring B and C N-H groups and, upon H/D exchange, shifts to the spectral region around 1070 cm^−1^. The band is observed in all variants, indicating that none of the amino acid substitutions affects the normal and fully protonated state of the cofactor. The other bands in this region are assigned to modes mainly including the C=C stretching coordinates of the methine bridges, as well as the C=O stretching of rings A and D (1720 cm^−1^). The weak H/D sensitivity of these modes results from the admixture of N-H in-plane bending coordinates of the adjacent pyrrole rings. The corresponding mode of the A-B methine bridge (A-B stretching) remains unchanged by the substitutions and is observed in the spectral region between 1650 cm^−1^ and 1656 cm^−1^ and downshifts to the region between 1646 cm^−1^ and 1649 cm^−1^ in D_2_O buffer. A similar position and H/D effect was observed for the near-infrared fluorescent RpBphP2-derived iRFP713 protein studied previously^24^. The latter study reported that the A-B stretching mode at a frequency above 1650 cm^−1^ is indicative of a strongly fluorescent conformer, in contrast to the weakly fluorescent species with an A-B stretching mode between 1640 and 1645 cm^−1^. The purified iRFP682(CC) shows the most upshifted mode at 1656 cm^−1^, indicating that the fluorescence is enhanced by a conformational change at the A-B methine bridge. This should result from the strongest stabilization of the ring A attached to two Cys.

In the spectral region between 1615 cm^−1^ and 1630 cm^−1^, two closely spaced modes are observed, involving the C=C stretching coordinates of the C-D methine bridge (C-D stretching) and of ring D in the low- and high-frequency component, respectively. The lower frequency mode at 1619 cm^−1^ in iRFP682(CS) also includes small admixtures (about 7%,^25^) of the N-H in-plane bending coordinates of rings C and D. Consequently, removal of these latter contributions upon H/D exchange causes a considerable downshift of this mode (C-D stretching) by 6 cm^−1^, in contrast to the nearly invariant high-frequency mode (ring D C=C stretching) such that the frequency separation between both modes increases. Unlike the ring D C=C stretching, the frequency of the C-D stretching mode displays considerable variations among the various species studied here. The lowest frequency at 1619 cm^−1^ is observed for iRFP682(CS) and (SS), whereas for all GAF-Cys containing variants, this mode is upshifted by nearly 10 cm^−1^ such that it coincides with the ring D C=C stretching. Therefore, for these iRFP variants, both modes are only separated in the spectra of the samples in D_2_O.

The proposed binding pattern for the double attachment (Fig. 1, structure D) lacks the endo- and exocyclic double bonds^11^. Since the stretching mode of the exocyclic DB, predicted to be above 1650 cm^−1^, is Raman-inactive^26^, the RR spectrum for the double-Cys attachment is expected to be very similar to that of the GAF-Cys-bound chromophore. In fact, the main peak in the C-D stretching region of iRFP682(CC) is observed at the same position as for the iRFP682(SC) variants at 1629 cm^−1^. However, no separated ring D C=C stretching peak is observed in iRFP682(CC) before or after purification, thus, single PAS-Cys attachment can be excluded for the double cysteine variant. This interpretation is supported by the UV/Vis spectra, because there is no indication of a 674-nm absorbance peak in the spectrum of the purified iRFP682(CC). The iRFP682(CC) “as isolated” spectrum comprises, in fact, a spectral superposition of iRFP682(SC) and the purified CC variant. The RR and the fluorescence spectra of iRFP682 “as isolated” can be reproduced as the sum of 45% double- and 55% GAF-only attached species (Supplementary Fig. S5).

## Conclusions

In this study, we report the purification and characterization of a bacteriophytochrome-derived fluorescent protein species containing an unusual double-Cys-attached chromophore for the first time. To isolate this variant, we applied a new purification protocol that takes advantage of the higher stability of the double-attached species against denaturants. Though this study was performed on the near-infrared fluorescent protein iRFP682 derived from RpBphP2^4^, our findings are applicable to other near-infrared fluorescent proteins that contain two chromophore-binding Cys residues in the PAS and the GAF domains, as the double-Cys-attached chromophores were detected in several proteins engineered from different BphPs^10,11^. UV/Vis absorption and fluorescence spectra or RR spectroscopic signatures, which are characteristic for distinct chromophore configurations, are identified. Importantly, the purified double-Cys-attached species is characterized by a FQY of 16.6%, the highest FQY reported for BphP-derived NIR FPs so far. Thus, together with the also increased extinction coefficient, the purified protein containing BV bound to two Cys simultaneously showed two-fold increased molecular brightness than the sample before purification (Table 1).

To improve brightness of future engineered iRFPs, the ratio of single- to double-Cys attachment needs to be shifted towards higher yield of double-attached chromophore. Assuming that the chemical reactions leading to covalent chromophore attachment are irreversible, this is a kinetic problem of two competing reaction pathways leading from free BV to either species C1 or D (Fig. 1). In iRFP682, the reaction kinetics of the two pathways are similar, resulting in an about 50:50 ratio of C1 and D. To increase the amount of species D, either the reaction A → D in Fig. 1 needs to be accelerated or A → C1 slowed down. These changes in reactivity could be accomplished by changing the electrostatic environment of the attachment site by further amino acid substitutions. Additionally, highly fluorescent chromophore states are characterized by a high RR signature band in the region around 1650 cm^−1^, which is characteristic for the ring A-B methine bridge C=C stretching coordinate and indicates rigid chromophore embedment, as concluded earlier^24^. This rigidity is most likely responsible for the exceptionally high FQY of BphP-derived NIR FPs containing two Cys residues. Analysis of the described spectroscopic features and denaturation behavior combined with rational mutagenesis of amino acids surrounding the two chromophore-binding Cys residues in the PAS and in the GAF domains should facilitate engineering of brighter NIR FPs.

## Materials and Methods

### Site directed mutagenesis, protein expression and purification

The cDNAs of the various iRFP constructs were subcloned into the pBAD plasmid vector (arabinose-inducible) and transformed into LMG194 *E. coli* cells (Thermo Fisher Scientific, Waltham, MA) that already harbored a plasmid carrying the cDNA of a heme oxygenase under the control of a rhamnose-inducible promoter, as described^10^. Site-directed mutagenesis was performed using the QuikChange II Site-directed mutagenesis kit (Agilent, Santa Clara, CA) according to manufacturer’s instructions. Oligonucleotides for mutagenesis were purchased from Eurofins MWG Operon (Ebersberg, Germany). All cDNAs were confirmed by sequencing (Eurofins MWG Operon). Protein expression in LMG194 *E. coli* cells and metal chelate affinity purification of the proteins was performed according to previously published procedures^10,24,27^. Analytical size-exclusion chromatography (SEC) used a Superdex 200 Increase 10/300 column (GE Healthcare Europe, Freiburg, Germany) according to manufacturer’s procedures.

### In-gel Zn^2+^ fluorescence

Protein samples were loaded at a concentration of 0.3 mg/ml (determined from the absorption at 280 nm calculated by ProtParam, https://web.expasy.org/protparam/) on a SDS gel and separated by electrophoresis prior to Zn^2+^ fluorescence. In-gel staining of biliverdin IXα bound to iRFP constructs was carried out as described^28^. The different fluorescence intensities of the samples treated with Zn^2+^ (Supplementary Fig. S1) are due to apoprotein contribution and incomplete chromophore assembly^10^. Densitometric analysis of protein bands in gel images was carried out with ImageJ.

### Spectroscopy

All measurements were performed under protective green light (502 nm). UV/VIS measurements were performed with a Cary E4 spectrophotometer (Agilent, Santa Clara, CA). Fluorescence measurements were performed using a Fluoromax 2 spectrometer (Horiba, Kyoto, Japan). Determination of the fluorescence quantum yield (FQY) was performed as described in the Supplementary Fig. S3. The excitation wavelength was set to 640 nm and the fluorescence signal was collected between 650 nm and 800 nm, and corrected according to the number of absorbed photons (absorbance at 640 nm). RR spectroscopic measurements were carried as described previously using a Fourier-transform (FT) Raman spectrometer with 1064-nm excitation^27^. All RR spectra shown in this work were measured at −140 °C, all UV/Vis and fluorescence spectra at room temperature (20 °C).

## Supplementary information

Chromophore binding to two cysteines increases quantum yield of near-infrared fluorescent proteins
