# Supplementary material for: Chromophore binding to two cysteines increases quantum yield of near-infrared fluorescent proteins
Source: Sci Rep. 2019 Feb 12;9:1866. doi: 10.1038/s41598-018-38433-2 (PMC6372600; doi:10.1038/s41598-018-38433-2)
Supplement: Supplementary file 1 — Chromophore binding to two cysteines increases quantum yield of near-infrared fluorescent proteins [file 41598_2018_38433_MOESM1_ESM.docx]

Supporting Information

**Chromophore binding to two cysteines increases quantum yield of near-infrared fluorescent proteins**

David Buhrke^1^, Neslihan N. Tavraz^1^, Daria M. Shcherbakova^2^, Luisa Sauthof^3^, Marcus Moldenhauer^1^, Francisco Vélazquez Escobar^1^, Vladislav V. Verkhusha^2^, Peter Hildebrandt^1^ and Thomas Friedrich^1^*

^1^ Institut für Chemie, Sekr. PC14, Technische Universität Berlin, Straße des 17. Juni 135, 10623 Berlin, Germany.

^2^ Department of Anatomy and Structural Biology, Albert Einstein College of Medicine, 1300 Morris Park Avenue, Bronx, NY 10461, USA.

^3^ Charité – Universitätsmedizin Berlin, Institute of Medical Physics and Biophysics (CC2), Group Protein X-ray Crystallography and Signal Transduction, Charitéplatz 1, 10117 Berlin, Germany.

* Correspondence: friedrich@chem.tu-berlin.de

**Contents**

1. Figure S1: SDS page and Zn^2+^ fluorescence
2. Figure S2: Absorbance curves during GdnHCl denaturation
3. Figure S3: Calculation of the fluorescence quantum yield
4. Figure S4: Full-range resonance Raman spectra
5. Figure S5: iRFP682(CC) as isolated is a mixture of species containing double-attached chromophore and species with chromophore attached to the GAF domain.
6. Figure S6: Original photographs of gel images shown in Figure S1 and Figure 2C
7. Literature cited

**
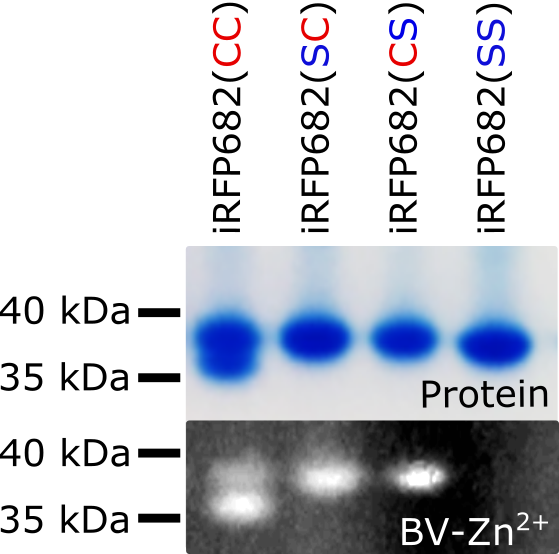
**

**Figure S1:** To investigate whether the chromophore was covalently attached to the respective apoproteins, SDS-PAGE with subsequent in-gel Zn^2+^ fluorescence assay was performed ^1^. Upper panel: Coomassie blue staining shows a single band for all protein variants containing one Cys. iRFP682(CC) displays a second band characteristic for iRFPs with a double-Cys motif. Lower Panel: Except for the iRFP682(SS) variant, the chromophore is covalently attached to the protein, as indicated by the characteristic fluorescence of the BV-Zn^2+^ complex. In-gel staining of biliverdin IXα bound to iRFP constructs was carried out as described ^1^. The concentration of the protein samples loaded on the gel was 0.3 mg/ml (determined from the absorption at 280 nm calculated by ProtParam).

**
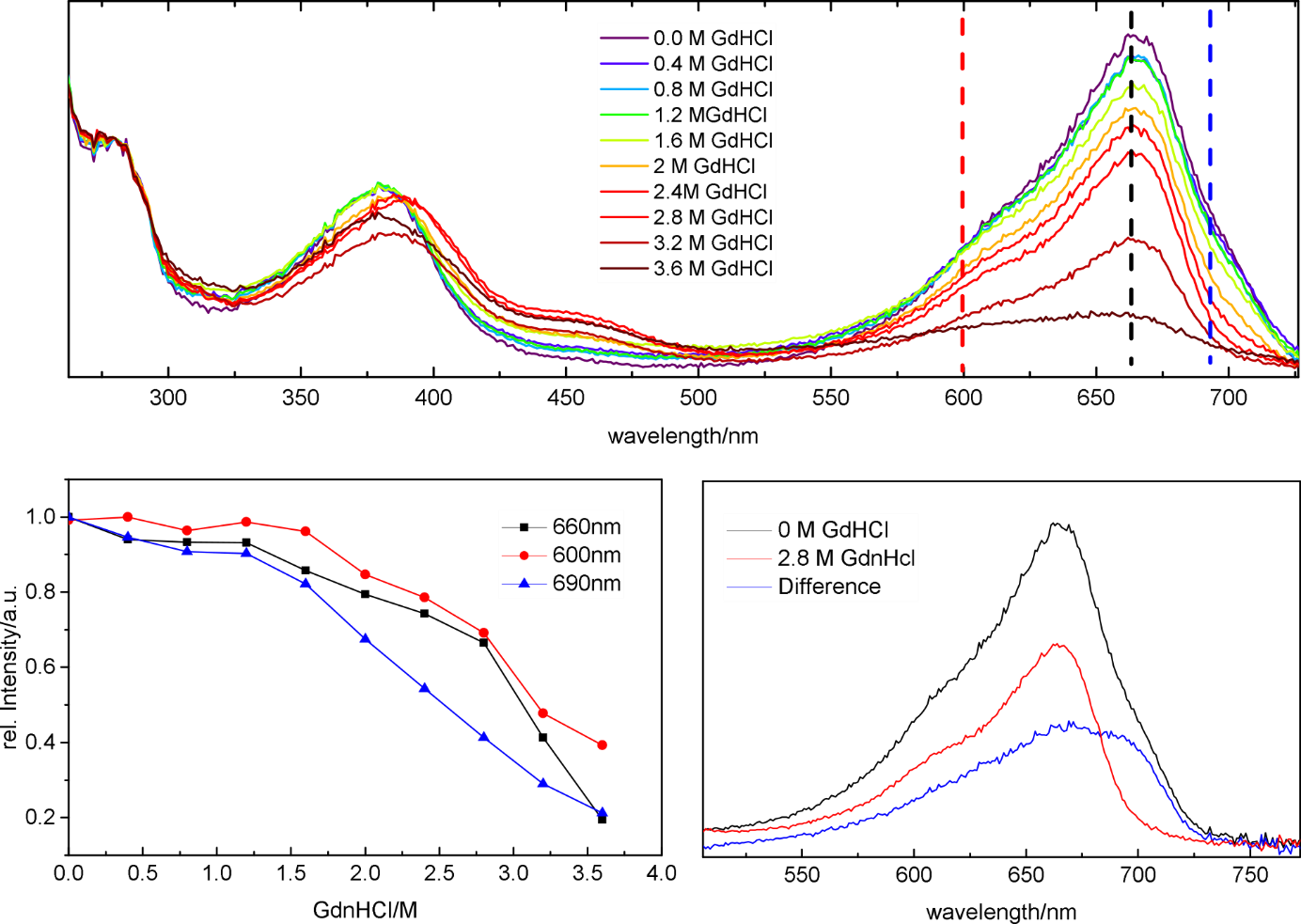
**

**Figure S2**: The affinity-purified iRFP682(CC) protein was titrated with GdnHCl as described previously^2^ and the heterogeneous denaturation behavior, affecting contributions of the protein species with different chromophore attachment differently (i.e. those hypothesized to be due to the single- and double-attached form, and small fraction of the species with non-covalently bound chromophore absorbing at 690 mn), was reproduced (upper panel). At 2.8 M GdnHCl, the deviation of the relative absorption intensities at 660 nm and 690 nm is the largest (lower left panel), indicating that the more stable double-attached species could be maximally enriched under these conditions, whereas the least stable form with non-covalently attached chromophore absorbing at 690 nm is denatured together with less stable single-attached form. Therefore, these conditions were chosen to induce denaturation of the single-attachment species and size-exclusion chromatography was performed subsequently in order to isolate the non-denatured (double-attached) species (see Fig. 1 in main article). The absorption spectra of the iRFP682(CC) “as isolated”, of the sample enriched in double-attached species after the denaturation purification scheme, and the difference spectrum (corresponding to the single-attached species lost during purification) is shown in the lower right panel.


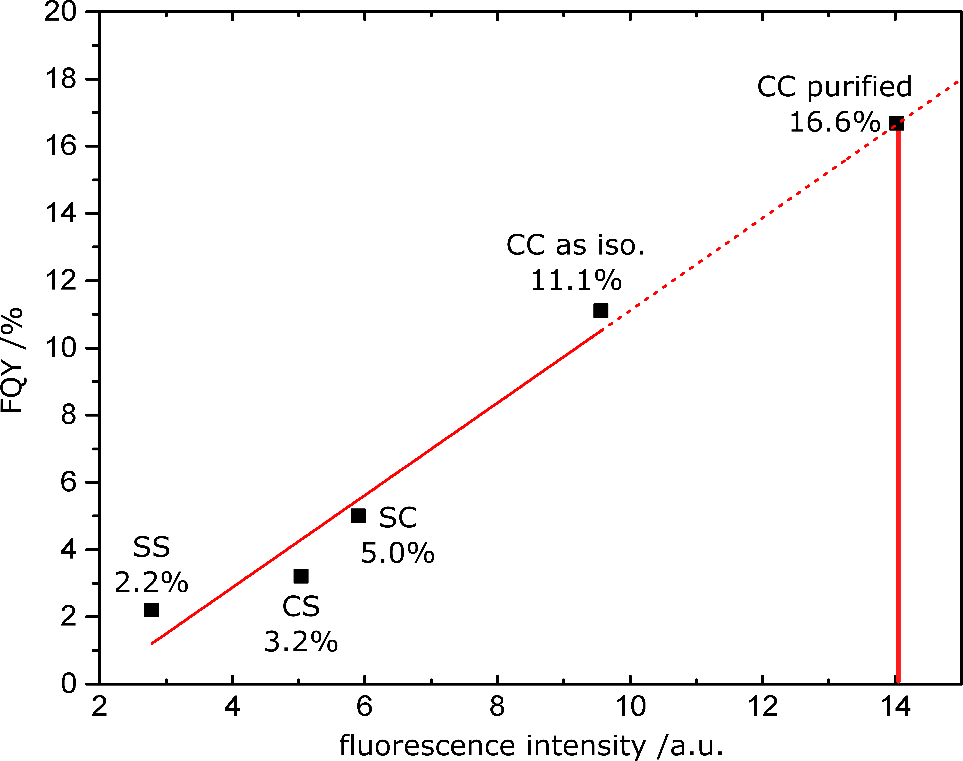


**Figure S3:** Calculation of the FQY of purified iRFP682(CC) from published data on the basis of FQY values determined for other protein variants in the literature (see Table 1 for references). The published FQY values were plotted against the experimentally determined integrated fluorescence intensities measured in a fluorescence spectrometer at defined protein concentrations (based on characteristic extinction coefficients). Linear extrapolation to the integrated fluorescence intensity measured for purified iRFP682(CC) yields an FQY of 16.6%.

**
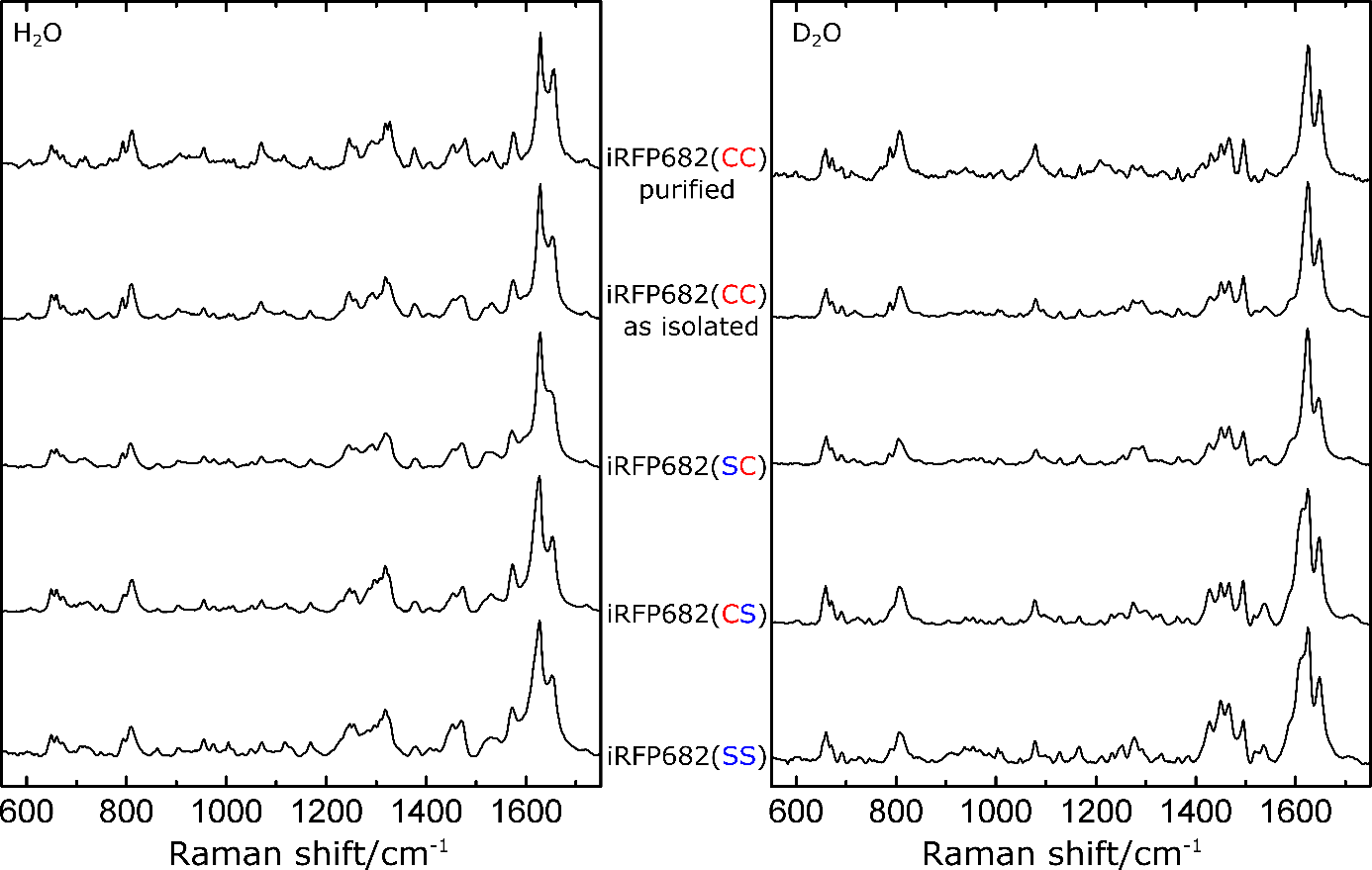
**

**Figure S4**: Full-range resonance Raman spectra of all investigated protein variants. The left panel shows measurements performed in H_2_O buffer, the right panel in D_2_O buffer.


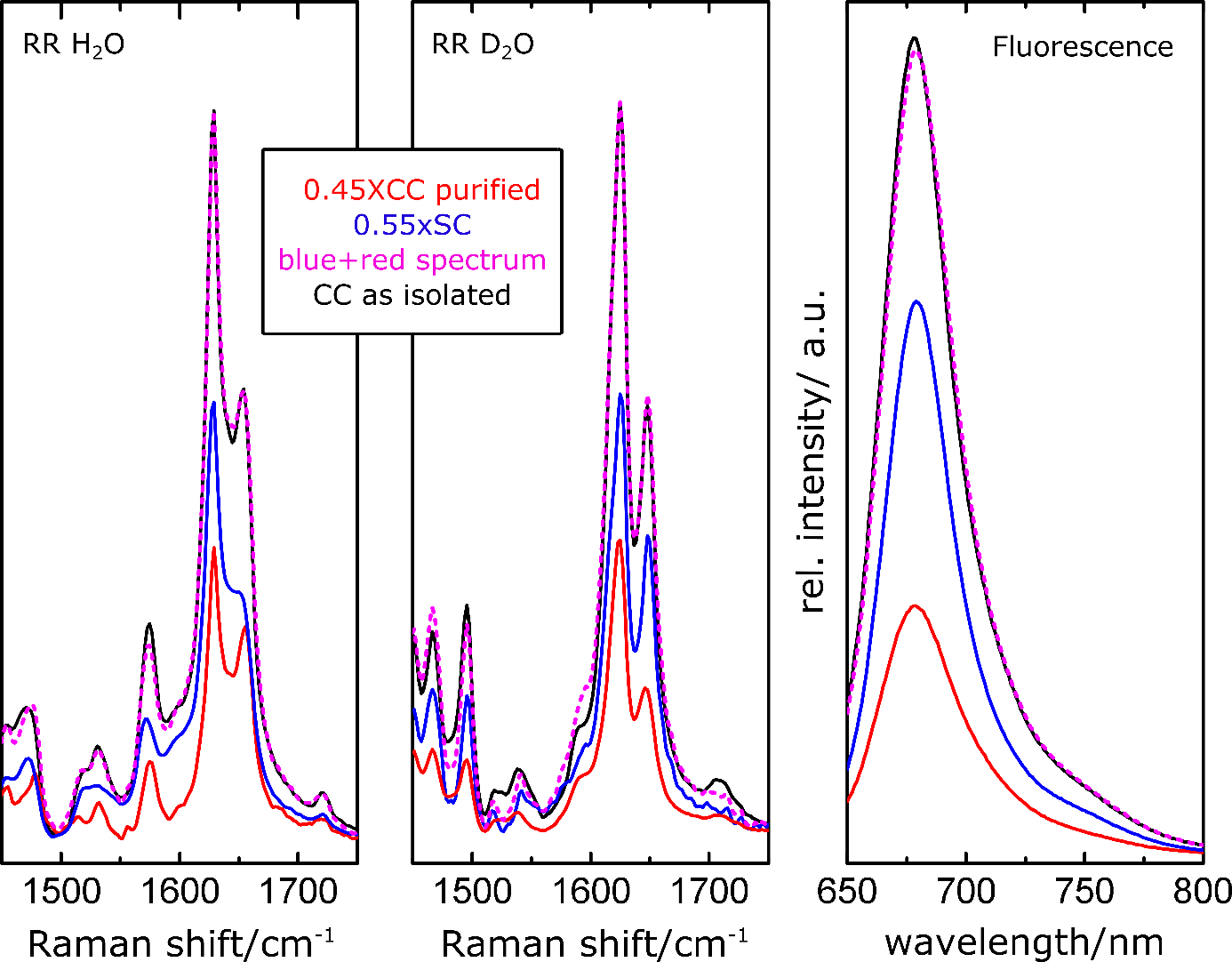


**Figure S5**: iRFP682(CC) as isolated is a mixture of species containing double-attached chromophore and species with chromophore attached to the GAF domain. The RR spectrum of IRFP682(CC) as isolated (black lines) represents a superposition of the pure double-attached species (red lines) and the single-attached species iRFP682(SC) (blue lines). The ratio of the spectral contributions is 0.45:0.55, as represented by the corresponding sum (magenta lines) of the spectra of single- and double-attached species, which matches the spectra of the “as isolated” species. Left panel: RR spectra in H_2_O, middle: in D_2_O. Right panel: fluorescence spectra according to the same color code.


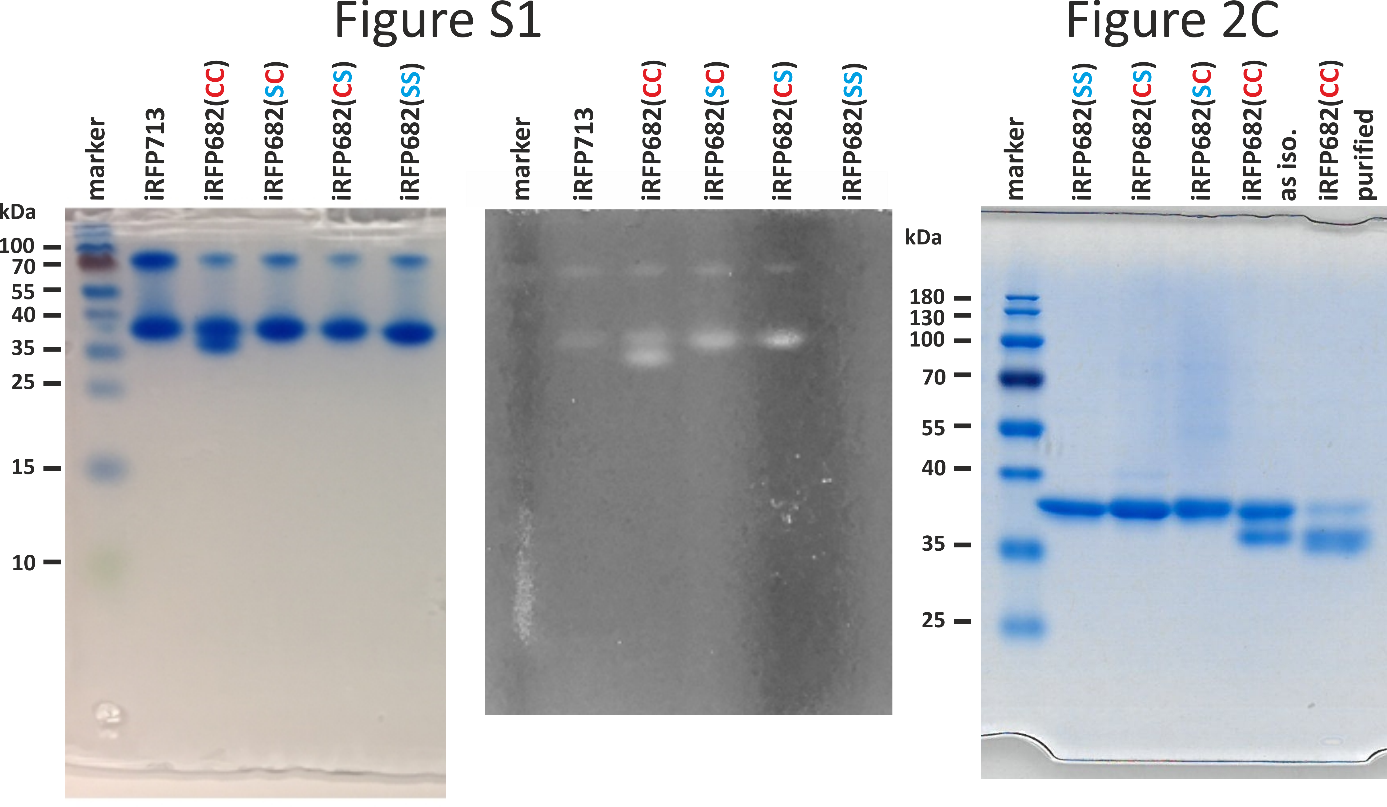


**Figure S6**: Original gel photographs of the Coomassie-stained SDS gel shown in Figure S1 (left panel), the corresponding in-gel Zn^2+^ staining fluorescence image (middle panel) and of the Coomassie-stained SDS gel shown in Figure 2C of the main manuscript.

**Literature cited:**

1 Raps, S. Differentiation between Phycobiliprotein and Colorless Linker Polypeptides by Fluorescence in the Presence of ZnSO(4). *Plant Physiol* **92**, 358-362 (1990).

2 Stepanenko, O. V. *et al.* Allosteric effects of chromophore interaction with dimeric near-infrared fluorescent proteins engineered from bacterial phytochromes. *Sci Rep* **6**, 18750, doi:10.1038/srep18750 (2016).
